# Dynamic Expression Profiles of β-Catenin during Murine Cardiac Valve Development

**DOI:** 10.3390/jcdd7030031

**Published:** 2020-08-17

**Authors:** Lilong Guo, Janiece Glover, Alyssa Risner, Christina Wang, Diana Fulmer, Kelsey Moore, Cortney Gensemer, Mary Kate Rumph, Reece Moore, Tyler Beck, Russell A. Norris

**Affiliations:** Department of Regenerative Medicine and Cell Biology, Medical University of South Carolina, Suite 601 Basic Science Building, 173 Ashley Avenue, Charleston, SC 29425, USA; guol@musc.edu (L.G.); gloverja@musc.edu (J.G.); risnera@musc.edu (A.R.); Christina.wang32@gmail.com (C.W.); fulmerd@musc.edu (D.F.); moorkels@musc.edu (K.M.); gensemer@musc.edu (C.G.); rumphm@musc.edu (M.K.R.); moorere@musc.edu (R.M.); beckt@musc.edu (T.B.)

**Keywords:** β-catenin, cardiac development, Lef-1, valve morphogenesis

## Abstract

β-catenin has been widely studied in many animal and organ systems across evolution, and gain or loss of function has been linked to a number of human diseases. Yet fundamental knowledge regarding its protein expression and localization remains poorly described. Thus, we sought to define whether there was a temporal and cell-specific regulation of β-catenin activities that correlate with distinct cardiac morphological events. Our findings indicate that activated nuclear β-catenin is primarily evident early in gestation. As development proceeds, nuclear β-catenin is down-regulated and becomes restricted to the membrane in a subset of cardiac progenitor cells. After birth, little β-catenin is detected in the heart. The co-expression of β-catenin with its main transcriptional co-factor, Lef1, revealed that Lef1 and β-catenin expression domains do not extensively overlap in the cardiac valves. These data indicate mutually exclusive roles for Lef1 and β-catenin in most cardiac cell types during development. Additionally, these data indicate diverse functions for β-catenin within the nucleus and membrane depending on cell type and gestational timing. Cardiovascular studies should take into careful consideration both nuclear and membrane β-catenin functions and their potential contributions to cardiac development and disease.

## 1. Introduction

β-catenin is a multifaceted protein with various functions, based on its subcellular localization [1,2,3,4,5]. Expression studies have demonstrated its presence on the cell membrane, free within the cytoplasm and in the nucleus [6,7,8,9,10]. Its subcellular functions are likely driven by conserved structural motifs within the protein, which confer unique protein–protein interactions [11,12,13]. The basic protein organization of β-catenin consists of an amino terminal domain, a central region consisting of twelve Armadillo repeats and a carboxyl-terminal region [14,15]. Through the Armadillo repeats, β-catenin serves as both a structural scaffold and signaling protein for a multitude of interaction partners in adherens junctions, the cytoplasm as well as the nucleus [16,17,18,19,20]. 

At the level of the membrane, β-catenin was initially discovered as being associated with E-cadherin, a critical protein essential for Ca^2+^-dependent cell adhesions [21,22,23]. Through these cadherin–catenin interactions, the adherens junctions becomes stabilized [24,25,26]. Upon receiving a Wnt signaling and/or phosphorylation of β-catenin at specific residues, this interaction is broken, and β-catenin is released from its junctional components [27,28,29]. The fate of β-catenin following this release is complex but likely results in either its cytoplasmic destruction or its nuclear import [30,31]. Although β-catenin does not contain an import or export signal, its nuclear presence can be driven by either protein chaperones or its binding to nuclear pore complexes [32,33,34]. Within the nucleus, β-catenin has been shown to regulate many aspects of nuclear biology. Most routinely analyzed are its ability to interact with co-transcription factors in the TCF/LEF1 gene family [35,36,37]. LEF1 plays the role of nuclear effector in the Wnt/β-catenin signaling pathway. In the absence of Wnt, LEF1 is bound by co-repressors that negatively regulate the expression of Wnt signaling genes. Following Wnt ligand stimulation, β-catenin displaces these transcriptional co-repressors and promotes LEF1 transcription factor activity to regulate a host of functions, including endothelial-to-mesenchymal transformation, proliferation and differentiation. The interactions between β-catenin and LEF1 have been historically viewed as inseparable. However, more recently, it has become clear that β-catenin can have unique TCF/LEF1 independent functions in the nucleus, including the regulation of chromatin remodeling as well as inducing or repressing gene transcription through association with other co-factors [38,39,40,41,42]. Through the complexities of the structural and signaling roles of β-catenin, it is no surprise that the perturbation of its expression can impact many different tissues in various ways. For example, gain and loss of function studies in the heart have revealed a critical role for β-catenin in cardiac development, especially related to valve morphogenesis [43,44]. 

**Figure 1:**
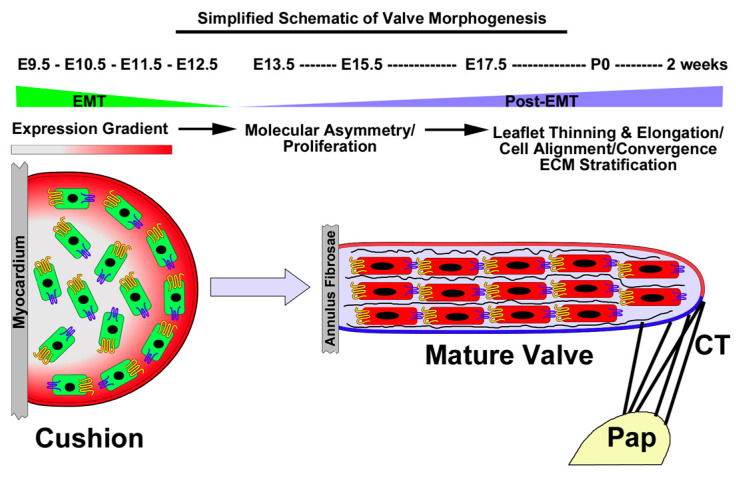
Simplified Schematic of Atrioventricular Valve Morphogenesis: A putative process of convergent extension. During E9.5-E12.5, the myocardium secretes various growth factors that result in an EMT reaction. Cells invade the underlying space and proliferate. Proliferation decreases during post-EMT along with the presence of molecular asymmetry of cadherins (yellow and blue membrane proteins). Cell density increases, resulting in cell–cell contact and intercellular organization and ECM/tissue stratification. The valve becomes attached to the papillary muscles (Pap) through chordae tendineae, which imparts tension on the tissue and facilitates leaflet thinning and elongation, reminiscent of convergent extension processes.

The fundamental aspects of valve development can be broadly characterized into two major morphogenetic events: Endothelial to Mesenchyme Transformation (EMT) and those events that follow EMT (called Post-EMT). The molecular and cellular processes that are defining characteristics of each of these two phases have been covered extensively in many reviews [45,46,47,48,49,50,51,52,53,54,55,56]. Briefly, during EMT, the myocardium releases various growth factors that promote a transformation of endocardium into invasive mesenchymal cells. This delamination is critical for valve morphogenesis and, at least initially, contributes the majority of cells to developing primitive valves (endocardial cushions). These newly formed mesenchymal cells appear randomly oriented within the cushion tissue, which is primarily comprised of proteoglycans and gradients of various growth factors. The hyaluronan-rich environment is a potent mitogen and mesenchymal cells undergo rapid proliferation. During and subsequent to proliferation, the maturing valve tissue enters a stage of post-EMT in which the valve endocardium becomes stabilized and proliferation subsides. Although much less is known about post-EMT valvulogenesis, recent studies focusing on genetic causes of mitral valve prolapse in humans have identified membrane receptors as critical for promoting post-EMT tissue re-organization. Although the mechanisms have not been completely uncovered, we propose that membrane proteins in the cadherin gene family (e.g., DCHS1) can function as intercellular molecular hooks at focal adhesions. Thus, proliferation is a pre-requisite for increased cell-density to allow cells to come together. Cell–cell contact results in changes in cytoskeleton and cellular alignment, which has been shown to occur during post-EMT in fetal chick valves [53]. This likely occurs in concert with increased mechanical tension, imparted by the forming collagenous mitral annular tissue, as well as the chordae tendineae. As a consequence, the valve tissue thins and elongates, not unlike a convergent extension process. Thus, while EMT is required for valve initiation, defects in this fundamental process are likely incompatible with life. However, molecular and cellular alterations that occur during post-EMT development may impact tissue shape and overall function and are anticipated as being compatible with life. As our work has previously focused on the role of the atypical cadherin, *DCHS1*, and how it can impact valve structure and function through stabilizing adherens junctions [57], we wished to explore the role of the well-studied adherens junction protein, β-catenin and determine whether its expression correlates to the above-described morphogenetic events (shown in Figure 1) during valve development.

Although heavily studied, a more thorough characterization of the expression and localization of either nuclear or membrane β-catenin throughout cardiac development has yet to be reported. Additionally, scant data have been shown that clarify the correlation of Lef1 and nuclear β-catenin at different gestation stages. This information is needed to inform various cardiovascular phenotypes due to its genetic perturbation. Our detailed analysis of β-catenin’s temporal, cell-specific subcellular protein expression pattern is the focus of this report. Herein we report a detailed β-catenin expression map within the heart, which will facilitate the proper interpretation of the gain and loss of functional data, as well as provide new insight into fundamental biological processes that are regulated by this important protein. 

## 2. Materials and Methods

Mouse husbandry and genotyping: Animals were kept in a 12-h light–dark cycle with food and water ad libitum. For all murine studies, a minimum of 3 mice were used at each stage of development. Each mouse was from a separate litter to account for any background variability. Data shown are representative images and stainings were consistent among each mouse analyzed at each particular timepoint. 

Immunohistochemistry (IHC) and fluorescence imaging: Immunohistochemical and fluorescence stains were performed on 5 μm paraffin-embedded sections from embryonic timepoints (E11.5, E13.5), fetal gestation (E17.5), neonatal (P0), and adult (6-month). Human mitral valve samples were processed and sectioned, as previously described [57]. Primary antibodies and their dilutions included: activated β-catenin (Cell signaling, #9566S, 1:100), total β-catenin (cell signaling, #9581S, 1:100), MF20 (Developmental Studies Hybridoma Bank, 1:50), PCAM-1 (Dianova, #DIA-310, 1:50), hyaluronan binding protein (HABP) (Calbiochem #385911, 1:100) and LEF1 (cell signaling, #2230S, 1:100). To determine the activated β-catenin distribution during valvulogenesis, we stained for β-catenin^pS552^. Both Akt and PKA were shown to phosphorylate β-catenin at Ser552 [58,59]. Phosphorylation at Ser552 induces β-catenin accumulation in the nucleus and increases its transcriptional activity [59,60,61]. The specificity of these antibodies were validated as recognizing the appropriate epitopes in various model systems, including knockout animals and cell lines [57,61,62,63,64,65]. Secondary antibodies were all purchased from Invitrogen, used at a 1:100 dilution, and included fluorophores 488 and 568. Nuclei were stained with Hoechst (Life Technologies, #H3569, 1:10,000). Slides were cover-slipped using Invitrogen SlowFade Gold Antifade Reagent (#S36936). Images were captured using Zeiss Axioscope M2.

## 3. Results and Discussion

### 3.1. β-Catenin Expression during Embryonic Cardiac Development

**Figure 2:**
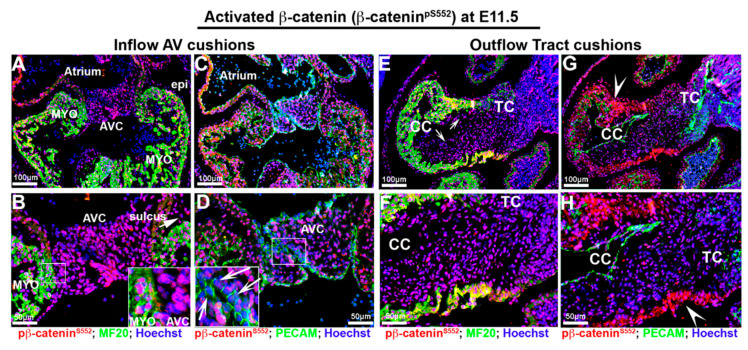
Activated β-catenin expression at E11.5 embryonic cardiac development. (**A**–**D**). Activated β-catenin (red) is ubiquitously expressed in the nuclei of virtually all cardiac cells including myocardium (myo), epicardium (epi), endothelium, sulcus tissue (arrow in (**B**)) aortic and atrioventricular cushion (AVC) cells. Staining intensity is consistently lower at the area where the superior and inferior AV cushions fuse ((**D**)—arrows), and the boundary between AV cushions and myocardium reflections ((**B**)—boxed region). (**E**–**H**) In the outflow segment, activated β-catenin is observed throughout the myocardium and outflow tract cushions ((**E**)—arrows—and (**F**)) Within the myocardial sleeve of the outflow tract, the cell membranes of myocytes were also stained positive for activated β-catenin ((**G**,**H**)—arrowheads). CC = conal cushions, TC = truncal cushion.

To determine activated and membrane β-catenin distribution during cardiac development, we stained for β-catenin^pS552^ and membrane β-catenin. Phosphorylation at Ser552 induces β-catenin accumulation in the nucleus and increases its transcriptional activity [59,60,61]. The specificities of these antibodies were validated as recognizing the appropriate epitopes in various model systems, including knockout animals and cell lines [57,61,62,63,64,65]. IHC staining for both activated (β-catenin^pS552^) and membrane β-catenin revealed dynamic staining patterns that are spatially and temporally regulated during embryonic gestation. At E11.5, activated β-catenin is ubiquitously expressed in the nuclei of virtually all cardiac cells, including myocardium, epicardium, endothelium and aortic and atrioventricular valve progenitor cells (Figure 2A–D). A slight, yet consistently lower IHC intensity of activated nuclear β-catenin expression was observed where the superior and inferior AV cushions fuse (Figure 2D—arrows). In the outflow segment, activated β-catenin is observed throughout the myocardium and outflow tract cushions (Figure 2E–H). Within the myocardial sleeve of the outflow tract, the cell membrane of myocytes also stained positive for activated β-catenin (Figure 2G,H—arrowheads). This staining seemed specific for the conotruncal myocardium, as no other myocardial regions within the heart showed membrane staining for activated β-catenin.

**Figure 3:**
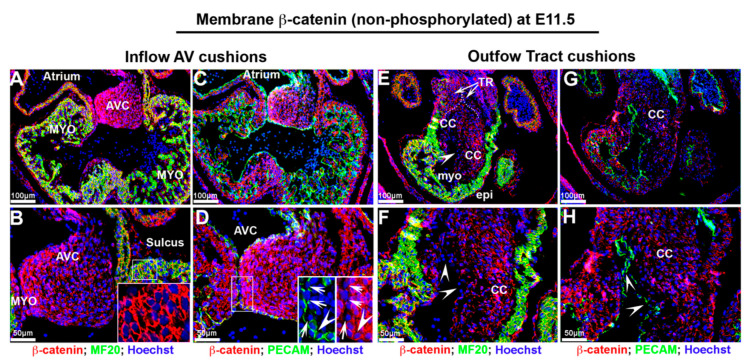
Non-phosphorylated β-catenin expression at E11.5 embryonic cardiac development. (**A**,**B**) Non-phosphorylated membrane β-catenin (red) was observed in developing atrioventricular cushions (AVC) with a gradient of staining intensity within the cushions. The myocardium (myo) exhibits robust membrane staining ((**B**)—boxed region). (**C**,**D**) Both Pecam and non-phosphorylated membrane β-catenin expression were dysregulated, and staining intensity was reduced at the area where the major cushions fuse ((**D**)—boxed region). (**E**,**F**) In outflow tract, membrane β-catenin displayed lower intensity in endocardium within conal cushions. (**G**,**H**) Pecam labeled endocardium showed lower to undetectable membrane β-catenin expression (arrowheads). TR = truncal ridges, CC = conal cushions.

Much like activated nuclear β-catenin expression, non-phosphorylated membrane β-catenin was also extensively observed throughout the heart at E11.5 (Figure 3A–H). Within the developing atrioventricular and outflow tract cushions, membrane β-catenin was observed in the endocardial and interstitial mesenchyme. Within these structures, the highest degree of intensity was observed nearest the cushion endocardium. However, in areas where the major cushions fuse, both PECAM and β-catenin expression appeared dysregulated and with reduced staining intensity (Figure 3D—arrowheads). This was similar to the pattern of membrane β-catenin in the conal cushions in which the endocardial epithelium displayed lower staining intensity (Figure 3E–G—arrowheads). Membrane expression of β-catenin was prominent in all cardiomyocytes as well as the ventricular endocardium and epicardium at this timepoint.

**Figure 4:**
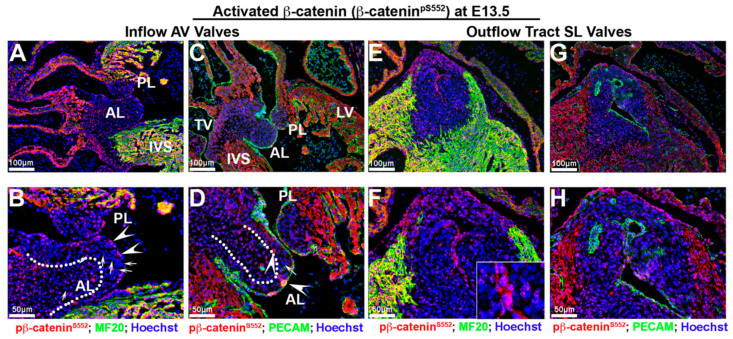
Activated β-catenin alters expression intensity and subcellular localization at E 13.5. (**A**–**D**) In atrioventricular valves, the expression of activated β-catenin (red) was reduced in valvular interstitial nuclei ((**B**,**D**)—arrows) and observed within the perinucleus and endocardial membrane ((**B**,**D**)—arrowheads). Nuclear β-catenin expression was primarily restricted to a group of cells within valve interstitial area ((**B**,**D**)—dotted line). (**E**–**G**). Downregulation and redistribution of activated nuclear β-catenin was also observed in the developing semilunar valves of the outflow tract. Within valve endocardial cells, activated β-catenin was present in nuclei as well as on the cell membrane ((**F**)—boxed region). Myocardium shared the same nucleolar and membrane β-catenin pattern as endocardium (**F**,**H**). AL = anterior leaflet, PL = posterior leaflet, IVS = interventricular septum, TV = tricuspid valve, SL = semilunar valves.

By E13.5, although expression within the mesenchymal cells of the AV valves is still evident, the expression of activated nuclear β-catenin appears reduced with evidence of perinuclear or membrane expression, especially within the AV valve endocardium (Figure 4B,D—arrowheads). Additionally, many interstitial cells within the AV valves failed to exhibit detectable activated β-catenin (Figure 4B,D—arrows). Valve interstitial cells closest to the AV valve endocardium appear to have lost or greatly downregulated the expression of activated β-catenin as nuclear expression is primarily restricted to a core group of cells within the valve (Figure 4B,D—dotted line). The downregulation of activated nuclear β-catenin was not specific to the AV valves, as we also observed this expression change in the developing semilunar valves of the outflow tract (Figure 4E–H). However, within the outflow tract valves, the expression of activated β-catenin was almost completely confined to the valve endocardium with only a few interstitial cells staining positive. Within valve endocardial cells, punctate nucleolar expression was evident as well as its presence on the cell membrane (Figure 4F—boxed region). Within the E13.5 myocardium, activated β-catenin was evident within the nuclei as well as on the cell membrane. Thus, within the heart at E13.5, a change in subcellular localization of activated β-catenin is evident, as compared to the E11.5 timepoint, whereby a nuclear-to-membrane shift of protein is observed. In addition, our stainings reveal a profound downregulation of the phosphorylated activated form of β-catenin within the developing mitral and aortic valves.

**Figure 5:**
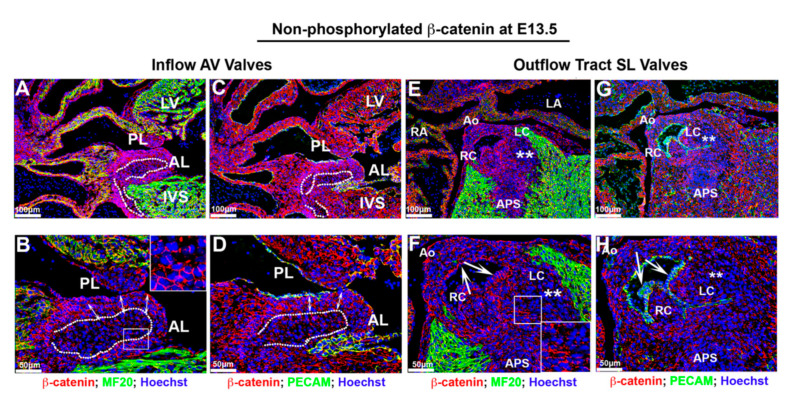
Membrane non-phosphorylated β-catenin at E13.5. (**A**–**D**) Membrane β-catenin expression was prominent in atrioventricular valves. However, within the central mass of valve interstitial cells ((**B**,**D**)—dotted line), the staining pattern was punctate and not continuous on the cell membrane as compared to cells in subendocardial region ((**B**,**D**)—arrows). (**E**–**H**) Membrane β-catenin displayed a similar spatial pattern as AV valves within the outflow tract mesenchyme of the semilunar valves. However, the majority of the interstitial mesenchyme in these valves presented a punctate pattern rather than circumferential β-catenin expression ((**F**)—boxed region). The outflow tract valve endocardium showed membrane expression of β-catenin along the basal aspect of these cells, which co-localized with the adherens junctions marker Pecam ((**F**,**H**)—arrows; Green: PECAM). Majority of interstitial cells within the left coronary cusp revealed very low to undetectable expression of β-catenin ((**E**–**H**)—asterisks). Outside of the valves, membrane expression of β-catenin was robust in all areas (cardiomyocytes, the epicardium, aortic wall and ventricular endocardium). AL = anterior leaflet, PL = posterior leaflet, IVS = interventricular septum, LV = left ventricle, RA = right atrium, Ao = aorta, APS = aorticopulmonary septum, RC =right coronary cusp, LC = left coronary cusp.

As our data showed reduced nuclear β-catenin activation at E13.5, we tested whether the expression of non-phosphorylated β-catenin showed a concurrent up-regulation and/or prominence at the cell membrane. As shown in Figure 5, membrane β-catenin was robust in all areas of the heart analyzed. The endocardium and subendocardial mesenchyme within the E13.5 atrioventricular (AV) valves displayed prominent membrane β-catenin staining. (Figure 5A–D). The graded staining pattern within the AV valves appeared similar to that observed at E11.5, albeit more pronounced at E13.5. The dotted lines in Figure 5A–D demarcate this unique spatial boundary between two apparently different cell phenotypes based on membrane stainings for β-catenin. Within the central mass of the valves, there appears to be a core of interstitial cells that display the uneven distribution of β-catenin staining. This is converse to the circumferential, tight junction appearing staining of the endocardial and subendocardial mesenchyme. Much like at E11.5 (Figure 3), this pattern of uneven distribution of staining possibly demarcates an interstitial cell type that is more primitive and/or shares a phenotype consistent with a less mature fibroblastic cell. A similar spatial pattern of β-catenin expression on the membrane is observed within the outflow tract mesenchyme of the semilunar valves. One noticeable difference is that the majority of the interstitial mesenchyme in these valves do not display circumferential β-catenin expression, but rather a punctate pattern (Figure 5F—boxed region). The outflow tract valve endocardium shows membrane expression of β-catenin along the basal aspect of these cells, consistent with the presence of adherens junctions (Figure 5F,H—arrows). Within the left coronary cusp, many of the interstitial cells had very low to undetectable expression of β-catenin (Figure 5E–H—asterisks). Outside of the valves, membrane expression of β-catenin was robust in all areas observed, including on cardiomyocytes, the epicardium, aortic wall and ventricular endocardium.

These expression data correlate with a timepoint of robust growth within the heart, consistent with a previously identified role for β-catenin in proliferation events. It is interesting to note that the endocardium of AV and conal cushion tissue that are destined to fuse at E11.5 show disorganized or reduced expression of both membrane and nuclear β-catenin, suggesting that the downregulation of the protein may be required for the differentiation of these cell types or may represent a consequence of compressive mechanical forces known to occur at these areas. Consistent with this concept of differentiation is the gradient of membrane expression observed within the AV cushions observed at E11.5 and E13.5. Alternatively, the difference in staining intensity could simply represent a different ultrastructural phenotype of the cell membrane within the valve interstitium. Previous reports have shown that subendocardial cushion mesenchyme are more densely packed along the atrialis compared to the more dispersed interstitial cells, proximal to the myocardium, thereby likely demarcating at least two different cell types within the AV cushions at this stage of development [53]. The difference in staining pattern and intensity within the core of the inflow and outflow tract valves at E13.5 further support this hypothesis.

### 3.2. β-Catenin Expression during Fetal Cardiac Development

**Figure 6:**
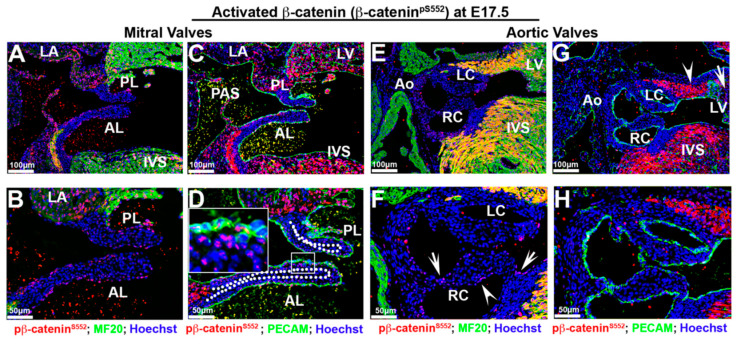
Activated β-catenin is downregulated in valvular tissues at E17.5 with non-uniform expression in myocardium. (**A**–**D**) Activated β-catenin was primarily observed in a subset of endocardial and subendocardial mesenchyme (boxed region in (**D**)). Most interstitial cells exhibited either undetectable or low levels of nuclear β-catenin expression. Regions outside of the mitral or aortic valves showed prominent nuclear β-catenin staining, including the primary atrial septum (PAS), the myocardial rim lining the mitral–aortic continuity, the left ventricular wall, the interventricular septum (IVS: interventricular septum), but the myocardium of the left atrium had much lower staining intensity than the adjacent left ventricular wall. (**E**–**F**). Activated β-catenin was only expressed in a few cells within the hinge regions connecting the aortic cusps to the aortic wall ((**F**)—arrows). Additionally, a few cells along one side of the right coronary cusp were positive for nuclear β-catenin expression ((**F**)—arrowheads) (**G**,**H**). Within the proximal/anterior heart regions, activated β-catenin expression was observed within the myocardial reflections adjacent to the aortic wall ((**G**)—arrowhead), whereas it was absent at basal myocardium ((**G**)—arrow). LC = left coronary cusp, RC = right coronary cusp, LV = left ventricle.

IHC was performed for both activated (β-catenin^pS552^) and non-phosphorylated β-catenin during fetal cardiac morphogenesis at E17.5. Within the mitral valves, activated β-catenin was observed in the nuclei of some cells in a spatial pattern similar to what was observed at E13.5 (Figure 4A–D and Figure 6A–D) with some slight, differences. Within the belly of the mitral leaflets, most interstitial cells exhibited either undetectable or low levels of β-catenin nuclear expression. Some, but not all, mitral valve endocardial and subendocardial interstitial cells displayed the weak expression of nuclear β-catenin (Figure 6B,D). This pattern of expression within the valves is reminiscent of the proteoglycan rich spongiosa region of the mitral valve. Co-immunostains of activated nuclear β-catenin with hyaluronan binding protein confirmed that nuclear β-catenin is primarily restricted to this particular valvular region at both E17.5 and E13.5 (Figure S1). Within the aortic valves, activated β-catenin was only observed in a few cells within the hinge regions connecting the aortic cusps to the aortic wall (Figure 6F—arrows). Additionally, a few cells along one side of the right coronary cusp were positive for nuclear β-catenin expression (Figure 6F—arrowhead). Thus, within the fetal mitral and aortic valves, our data would support a continual, gradual downregulation of nuclear β-catenin during gestation.

Regions outside of the mitral or aortic valves showed prominent nuclear β-catenin staining, including the primary atrial septum, the myocardial rim lining the mitral–aortic continuity and the inter ventricular septum (Figure 6A–G). The myocardium of the left atrium also appears to be positive, albeit at a much lower staining intensity than the adjacent left ventricular wall (Figure 6C). Within the left ventricular wall, we noticed that nuclear β-catenin within the distal/posterior basal myocardium showed robust staining, yet in more proximal sections this staining was largely absent (Figure 6G—arrow). Within the proximal/anterior heart regions, expression is observed within the myocardial reflections adjacent to the aortic wall (Figure 6G—arrowhead). These data suggest that β-catenin expression within the left ventricular myocardium is non-uniform and may demarcate either different cell populations or spatially dependent functional requirements for these cells.

**Figure 7:**
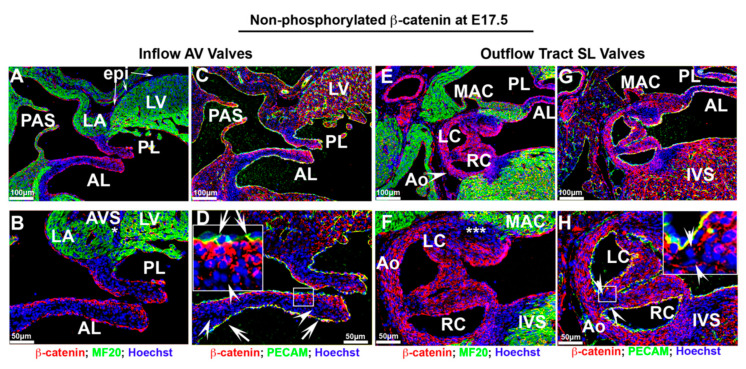
Membrane β-catenin expression at E17.5 fetal cardiac development. (**A**–**D**). Membrane β-catenin was expressed prominently throughout the valve endocardium within the anterior, posterior mitral leaflets, ventricular endocardium and myocardium. Cells sub-adjacent to the atrialis endocardium of the mitral leaflets displayed positive staining with much less to undetectable expression within the rest of the mitral leaflets ((**D**)—arrowheads). Membrane β-catenin was undetectable on epicardial cells within the left ventricle or within the atrioventricular sulcus ((**A**,**B**)—asterisk). (**E**–**H**) Within the aortic valves, membrane β-catenin was evident on all endocardial cells and most interstitial cells as well as within the mitro–aortic continuity (MAC). A subset of cells within aortic wall adjacent to MAC had undetectable staining ((**F**)—asterisks). Membrane staining was not observed on interstitial cells within the hinge regions ((**H**)—boxed region).

The staining for membrane bound, non-phosphorylated β-catenin at E17.5 exhibited a much different pattern than observed for the nuclear form, especially within the aortic valves (Figure 7). Within the anterior and posterior mitral leaflets, membrane β-catenin was expressed prominently throughout the valve endocardium (Figure 7A–D—arrows). Cells sub-adjacent to the atrialis endocardium of the mitral leaflets displayed positive staining with much less to undetectable expression within the rest of the mitral leaflets (Figure 7D—arrowheads). Within the aortic valves, widespread membrane β-catenin was evident on all endocardial cells and most interstitial cells (Figure 7E–H). Unlike the activated form of β-catenin, low to undetectable membrane staining was evident on interstitial cells within the hinge regions (Figure 7H—boxed region). Unlike the mitral valve, the staining within the aortic valve does not appear to be confined to one particular cell layer within the valve cusps. Outside of the mitral and aortic valves, membrane expression is observed in most cell types including ventricular endocardium and myocardium and on myocytes within the mitro–aortic continuity (MAC) (Figure 7E–G). Membrane β-catenin was undetectable on epicardial cells within the left ventricle or within the atrioventricular sulcus (Figure 7A,B—asterisk).

### 3.3. Postnatal Cardiac β-Catenin Expression

**Figure 8:**
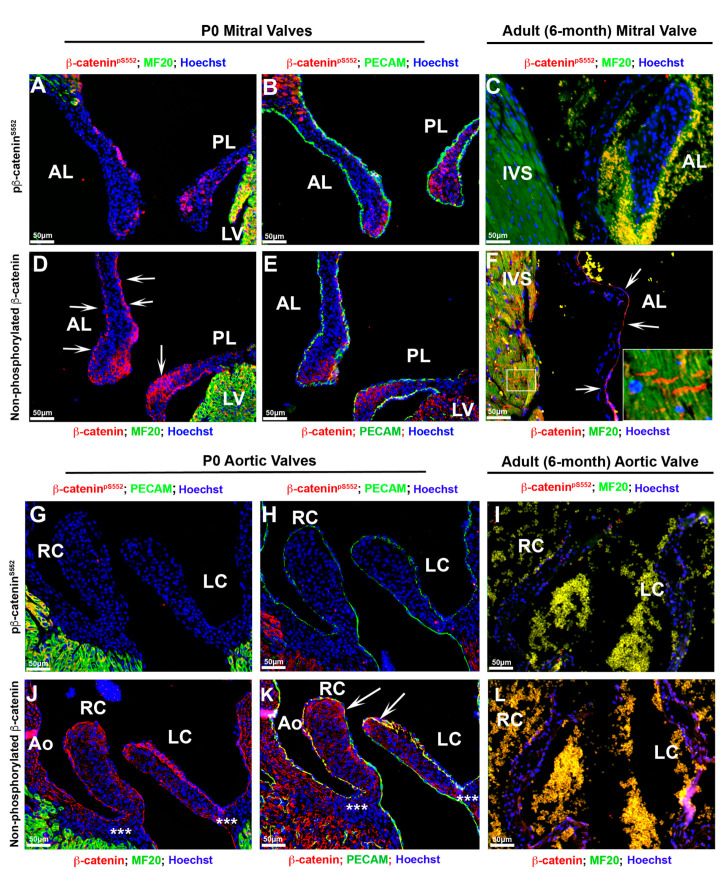
Postnatal cardiac β-catenin expression. (**A**,**B**) Both forms of β-catenin were stained on P0 and 6-month adult tissues. Activated form of β--catenin was only present within a subset of mitral valve interstitial cells confined to the tips at neonatal timepoints; however, the mitral valve endocardium is mostly devoid of positive nuclear staining. (**D**,**E**) Membrane staining for β-catenin was observed on mitral valve endocardium and co-labeled with Pecam ((**D**)—arrows—and (**E**)). (**C**,**F**) In adult mice no detectable nuclear β-catenin was observed within the mitral leaflets and IVS myocardium (**C**), whereas the non-phosphorylated β-catenin isoform was present primarily along the valve endocardial lining of the atrialis ((**F**)—arrows) and within the intercalated discs of cardiomyocytes ((**F**)—boxed region). (**G**,**H**) Activated form of β-catenin was not detectable within the aortic valves at neonatal time point. (**J**,**K**) Membrane-bound β-catenin co-labeled with Pecam within the aortic valve endocardium as well as a subset of interstitial cells at the tips of the cups ((**K**)—arrows). Membrane bound β-catenin was not observed within the hinge regions of the aortic cups (asterisks in (**J**)), but was found within the aortic wall (**J**,**K**). (**I**,**L**) Neither form of β-catenin was observed in the adult aortic valve leaflets. AL = anterior leaflet, PL = posterior leaflet, IVS = interventricular septum, LV = left ventricle, Ao = aorta, RC = right coronary cusp, LC = left coronary cusp.

As either loss of or gain of function of β-catenin has been shown to contribute to cardiac valvular diseases and postnatal cardiac regenerative processes, we sought to evaluate the expression of β-catenin after birth. As shown in Figure 8, activated β-catenin is only present within a subset of mitral valve interstitial cells confined to the tips at neonatal timepoints. The mitral valve endocardium is mostly devoid of positive nuclear staining (Figure 8A,B). On the contrary, membrane staining for β-catenin is robust in the mitral valve endocardium and co-labels with CD31/Pecam at adherens junctions (Figure 8D,E). In adult mice no detectable nuclear β-catenin is observed within the mitral leaflets whereas the non-phosphorylated β-catenin isoform is present primarily along the valve endocardial lining of the atrialis (Figure 8C,F). Consistent with previous reports, nuclear β-catenin is undetectable within the IVS myocardium (Figure 8C), but the non-phosphorylated isoform is observed within the intercalated discs of cardiomyocytes (Figure 8F—boxed region). The aortic valves show a similar pattern for β-catenin expression compared to the mitral valves, with one exception (Figure 8G–L). While membrane bound β-catenin co-localizes with CD31/pecam within the aortic valve endocardium, as well as a subpopulation of interstitial cells at the tips of the cusps (Figure 8J,K—arrows), we fail to detect the presence of nuclear β-catenin within the aortic valves (Figure 8G,H). Similarly, neither form of β-catenin is observed in the adult aortic valve leaflets (Figure 8I,L). At the postnatal timepoints we do not observe β-catenin expression within the hinge regions of the aortic cusps, but do observe membrane staining within the aortic wall (Figure 8J,K—asterisks). These data demonstrate that activated nuclear β-catenin expression is only detectable during a developmental and early neonatal window, whereas membrane-bound β-catenin continues throughout life, at least in the atrial aspect of the mitral valve.

### 3.4. Correlation of β-Catenin Activities with Lef1

**Figure 9:**
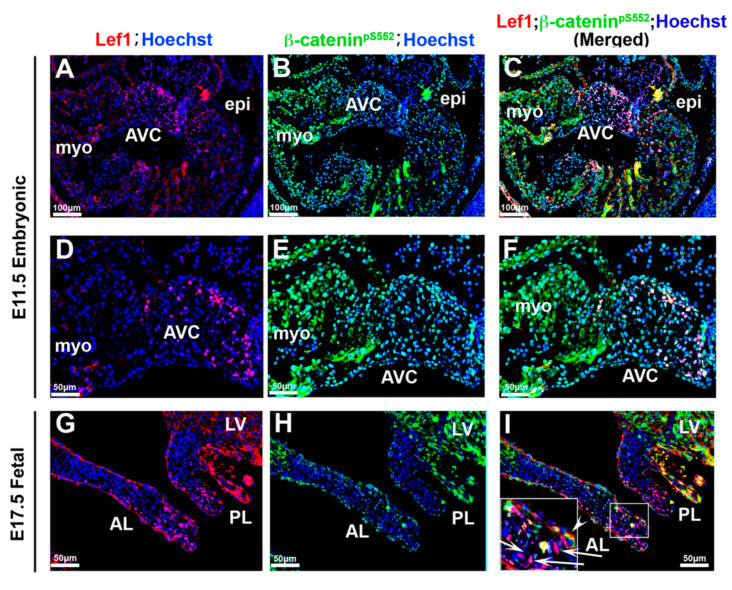
Correlation of β-catenin activities with Lef1 (**A**–**F**). Activated β-catenin (green) and Lef1 (red) were co-stained on E11.5 tissue. Only a subset of activated β-catenin positive cells were co-stained with Lef1. (**G**–**I**) Co-staining Lef1 with activated β-catenin at E17.5. Lef1 positive cells were localized to the tip of the leaflets showing no appreciable β-catenin staining ((**G**,**I**)—arrows) and valve endocardial cells that co-stained with both markers were rarely observed ((**I**)—arrowhead). AVC = atrioventricular cushions, myo = myocardium, epi = epicardium, AL = anterior leaflet, PL = posterior leaflet, LV = left ventricle.

Activated β-catenin can enter the nucleus and interact with members of the TCF/LEF transcription factor gene family to activate the transcription of downstream target genes [26,35,66]. Therefore, Lef1 is commonly used as a readout marker for β-catenin signaling [67,68,69,70,71,72]. More recently, the existence of β-catenin-independent functions for Lef1 have been documented, bringing into question whether lef1 reporters represent physiologically accurate measures of β-catenin signaling. To test this concept, we co-stained activated β-catenin with Lef1 on mitral valve tissues during embryonic and fetal gestation to determine if they co-localized with each other (Figure 9). During embryonic timepoints, most of the Lef1 positive stained cells were co-stained with nuclear β-catenin. However, a major discrepancy at this timepoint was that Lef1 was positive only in a subset of cells, indicating a lef1-independent nuclear role for β-catenin. The staining at E17.5 showed an even more striking discrepancy between Lef1 and nuclear β-catenin staining. Lef1 positive cells were observed within the nuclei of mitral valve interstitial cells localized to the tip of the leaflets. However, these positively stained Lef1 cells showed little detectable β-catenin staining (Figure 9I—arrows). We rarely observed valve endocardial cells that co-stained with both markers (Figure 9I—arrowhead). These data support the contention that, although there are a small subset of cells that co-express Lef1 and β-catenin, the majority of their activities are not mutually inclusive; likely representing independent roles for these proteins during cardiac valve development.

### 3.5. Human Myxomatous Mitral Valves Have Increased Nuclear β-Catenin

**Figure 10:**
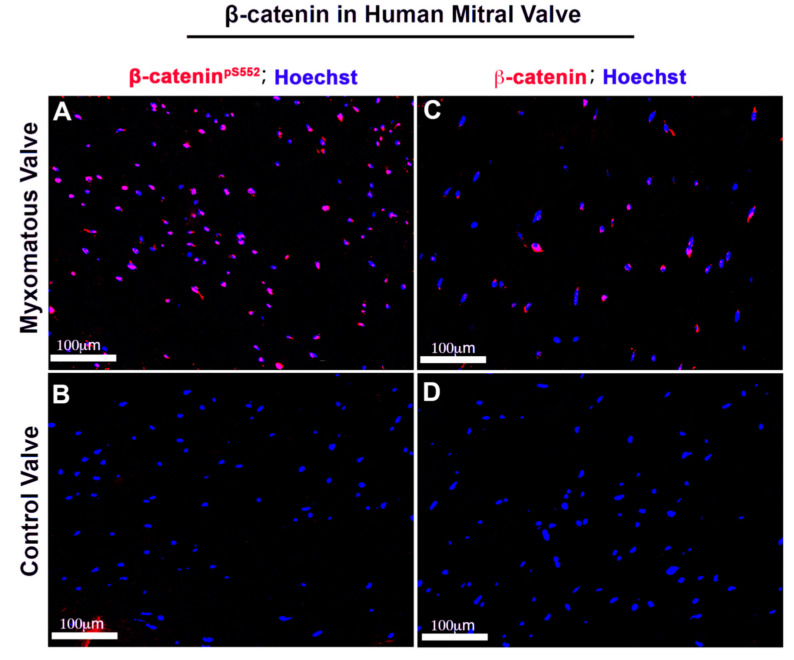
Human myxomatous mitral valves have increased nuclear β-catenin. Human adult myxomatous mitral valve (**A**,**C**) and control mitral valve tissues (**B**,**D**) were stained for activated and membrane β-catenin (red). Control adult mitral valve leaflets had undetectable activated or membrane β-catenin expression. Myxomatous valve tissue had increased activated and non-phosphorylated β-catenin compared to the control.

To determine the importance of β catenin signaling in human myxomatous valves, activated and membrane bound β-catenin antibodies were used to stain human valve tissues. Similar to what was observed in murine adult tissues (Figure 8C,F), unaffected human valve tissue had undetectable β-catenin expression. However, the human myxomatous valve had increased expressions of both activated and membrane-bound β-catenin, suggesting a significant increase in its expression (Figure 10).

### 3.6. Conclusions and Perspectives

β-catenin is a member of the armadillo family of proteins, which plays a significant role in cadherin-based cell–cell adhesion and is an indispensable co-activator of Wnt-mediated gene expression [35,73,74,75]. The dynamic regulation of its subcellular distribution, driven by phosphorylation and/or dephosphorylation events likely dictate its versatile functions [76]. Of note, the phospho-antibody used in this study recognizes a kinase event at β-catenin serine 552 that is driven by AKT and/or PKA. Although the function of these two signaling factors has been extensively studied in other tissues, their role in cardiac morphogenesis and valvulogenesis has not been thoroughly investigated. The phosphorylation of β-catenin can also occur at other residues by various upstream kinases, including src, EGFR, GSK, CK1 and CK2. However, in our hands, antibodies directed against these other phospho-sites provided inconsistent findings in IHC and were unable to be verified in knockout model systems.

Within the membrane, β-catenin is a critical component of the adherens junction, which helps stabilize intercellular interactions [25]. This Ca^2+^-dependent cell–cell adhesion event is fundamental for regulating morphogenetic processes, such as endocardial stabilization post-EMT [77,78]. The function of β-catenin at the membrane is distinct from its unique transcriptional-regulating properties within the nucleus [79]. Of note, we propose that care should be given in interpreting Lef1 data and/or reporter systems that use Lef1 as a readout for β-catenin nuclear activities. Our data demonstrate that the co-expression of Lef1 with nuclear β-catenin is minimal within the heart. It remains possible that β-catenin is interacting with other TCF factors within the heart. Indeed, our RNAseq datasets demonstrate that each of the other TCF factors (TCF7, TCF7L1 and TCF7L2) are expressed within the mitral valve at P0 and whole heart at E13.5, although Lef1 is the highest expressed, based on transcript counts [80]. Future studies should reveal whether these additional factors are co-expressed and interact with β-catenin and are needed to activate or repress target gene transcription. Regardless, our expression studies support a very early embryonic role for nuclear β-catenin, which correlates with active growth and proliferation of the heart. As the heart matures, a reduction in nuclear expression coincident with known timepoints of reduced proliferation were observed [81]. Previous studies have revealed that the loss of β-catenin or the β-catenin antagonist, Axin2, can result in profound valvulopathies in mice [44,82]. Herein we also show that nuclear β-catenin is increased in human myxomatous mitral valves from MVP patients. Whether this reflects a primary cause of MVP in humans or is a consequence of a disease phenotype, remains unknown. Our data demonstrate that activated nuclear β-catenin correlates with proliferation states during embryonic development. Thus, it remains plausible that a myxomatous valve reactivates nuclear β-catenin, resulting in VIC hyperplasia, a likely contributor to valve disease. Based on our detailed subcellular expression maps for β-catenin, it seems likely that the origin of some valve disease phenotypes stem, at least partially, from altered intercellular communications, and not solely due to the altered nuclear presence of β-catenin. If true, this concept would be consistent with other findings in the mitral and aortic valve that have linked disease phenotypes to altered cell–cell interactions, including recent studies on the cadherin proteins DCHS1 [57] and CAD-11 [83,84,85]. Thus, we posit that understanding how cadherin biology and intercellular interactions drive valve morphogenesis at the level of the membrane may reveal new mechanisms of development that are likely to be relevant to human disease phenotypes.

## Supplementary Materials

The following are available online at https://www.mdpi.com/2308-3425/7/3/31/s1, Figure S1: Activated β-catenin is prominent in proteoglycan enriched region within developing mitral leaflets. (A,B). At E13.5 and E17.5, activated β-catenin (red) expression is enriched within proteoglycan rich regions as detected by hyaluronan acid binding protein (HABP) (green), becoming restricted to the spongiosa region of the mitral valve as shown by the dotted lines and boxed region.
