# Supplementary material for: Dynamic Expression Profiles of β-Catenin during Murine Cardiac Valve Development
Source: J Cardiovasc Dev Dis. 2020 Aug 17;7(3):31. doi: 10.3390/jcdd7030031 (PMC7570242; doi:10.3390/jcdd7030031)
Supplement: Supplementary file 1 [file jcdd-07-00031-s001.pdf]

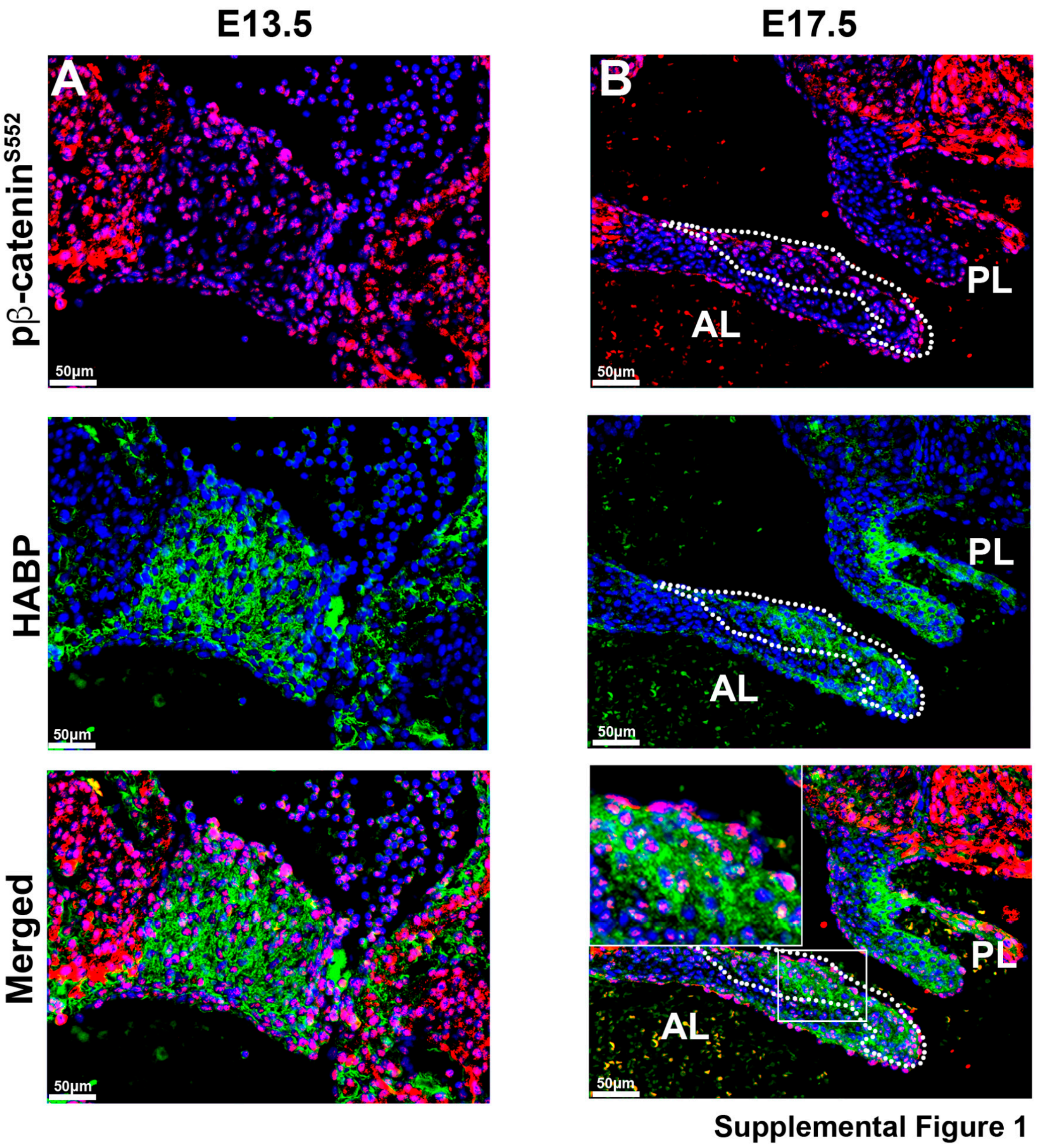

Supplementary Figure 1. Activated β-catenin is prominent in proteoglycan enriched region within developing mitral leaflets. (**A–B**). At E13.5 and E17.5, activated β-catenin (red) expression is enriched within proteoglycan rich regions as detected by hyaluronan acid binding protein (HABP) (green), becoming restricted to the spongiosa region of the mitral valve as shown by the dotted lines and boxed region.
